# Current status and applications of genome-scale metabolic models

**DOI:** 10.1186/s13059-019-1730-3

**Published:** 2019-06-13

**Authors:** Changdai Gu, Gi Bae Kim, Won Jun Kim, Hyun Uk Kim, Sang Yup Lee

**Affiliations:** 1 Department of Chemical and Biomolecular Engineering (BK21 Plus Program), Metabolic and Biomolecular Engineering National Research Laboratory, Institute for the BioCentury, Korea Advanced Institute of Science and Technology (KAIST), Daejeon, 34141 Republic of Korea; 2 Department of Chemical and Biomolecular Engineering (BK21 Plus Program), Systems Biology and Medicine Laboratory, KAIST, Daejeon, 34141 Republic of Korea; 3 Systems Metabolic Engineering and Systems Healthcare Cross-Generation Collaborative Laboratory, KAIST, Daejeon, 34141 Republic of Korea; 4 BioProcess Engineering Research Center and BioInformatics Research Center, KAIST, Daejeon, 34141 Republic of Korea

## Abstract

Genome-scale metabolic models (GEMs) computationally describe gene-protein-reaction associations for entire metabolic genes in an organism, and can be simulated to predict metabolic fluxes for various systems-level metabolic studies. Since the first GEM for *Haemophilus influenzae* was reported in 1999, advances have been made to develop and simulate GEMs for an increasing number of organisms across bacteria, archaea, and eukarya. Here, we review current reconstructed GEMs and discuss their applications, including strain development for chemicals and materials production, drug targeting in pathogens, prediction of enzyme functions, pan-reactome analysis, modeling interactions among multiple cells or organisms, and understanding human diseases.

**Electronic supplementary material:**

The online version of this article (10.1186/s13059-019-1730-3) contains supplementary material, which is available to authorized users.

## Introduction

Since the first genome-scale metabolic model (GEM) of *Haemophilus influenzae* RD was reported in 1999 [1], GEM reconstruction has been established as one of the major modeling approaches for systems-level metabolic studies. A GEM computationally describes a whole set of stoichiometry-based, mass-balanced metabolic reactions in an organism using gene-protein-reaction (GPR) associations that are formulated on the basis of genome annotation data and experimentally obtained information [2, 3]. Importantly, the GEM allows the prediction of metabolic flux values for an entire set of metabolic reactions using optimization techniques, such as flux balance analysis (FBA), which uses linear programming [4]. GEM also serves as a platform for the integration and analysis of various types of data such as omics and kinetic data [5–7]. As the techniques for genome sequencing and relevant omics analyses continue to evolve, the quality and application scopes of GEMs have also expanded accordingly, and together they have contributed to better understanding of metabolism in various organisms. Starting with GEMs of model organisms, including *Escherichia coli* [8] and *Saccharomyces cerevisiae* [9], GEMs of various microorganisms and also multicellular organisms, such as humans [10] and plant cells [11], have been reconstructed.

**Fig. 1:**
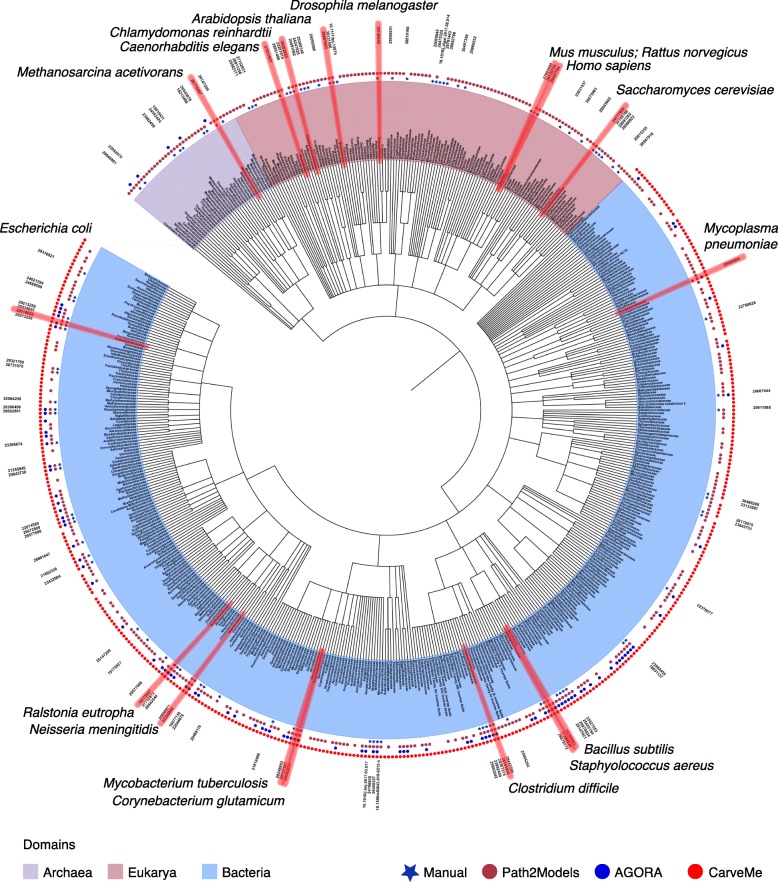
A phylogenetic tree of all of the GEMs reconstructed to date at the family level. GEMs for 434, 40, and 117 taxonomic families of bacteria (*light blue*), archaea (*light purple*), and eukarya (*pink*), respectively, are marked in the phylogenetic tree. Organism names are labeled with *circles* of different colors outside the circular phylogenetic tree, depending on the development methods used: manual, Path2Models [13], AGORA [14], and CarveMe [15]. For manually reconstructed GEMs, the relevant PubMed identifier (PMID) or digital object identifier (DOI) for the latest GEM version is additionally indicated. The phylogenetic tree was prepared as follows. First, organism names were collected from BioModels for the GEMs from Path2Models, from Virtual Metabolic Human (VMH) for AGORA models, and from a GitHub repository (https://github.com/cdanielmachado/embl_gems/blob/master/model_list.tsv) for CarveMe. Next, *National Center for Biotechnology Information* (NCBI) taxids at the species level and taxonomic lineages for all of the organisms subjected to the GEM reconstruction were obtained from a dataset available (as of May 14, 2019) at the NCBI Taxonomy FTP (ftp://ftp.ncbi.nih.gov/pub/taxonomy/taxdump.tar.gz). Finally, a Newick file for all of the organisms with taxids was subsequently generated using an in-house Python script at the family level, and this file was used to create a phylogenetic tree using iTOL (https://itol.embl.de/) [16]. A phylogenetic tree of GEMs at the species level is available as Additional file 1. A full list of organisms subjected to the GEM reconstruction and preparation of phylogenetic trees is available as Additional file 2

Such progress in the reconstruction of GEMs has made it possible to construct a wide range of metabolic studies by generating model-driven hypotheses and implementing various context-specific simulations [12]. Relevant applications that have benefited from advances in the use of GEMs include, but are not limited to, strain development for the production of bio-based chemicals and materials, drug targeting in pathogens, the prediction of enzyme functions, pan-reactome analysis, modeling interactions among multiple cells or organisms, and understanding human diseases. Applications of GEMs are expected to expand further in coming years. To this end, we comprehensively review the current status and applications of GEMs reconstructed for diverse organisms belonging to bacteria, archaea, and eukarya (Fig. 1, Additional file 1, and Additional file 2). By discussing a broad range of studies involving GEMs, we show how GEMs can help us to gain novel biological insights beyond those provided by genome sciences and how they can help to develop biotechnological applications.

## Current status of reconstructed genome-scale metabolic models

As of February 2019, GEMs have been reconstructed for 6239 organisms (5897 bacteria, 127 archaea, and 215 eukaryotes), either manually or by using automatic GEM reconstruction tools that are discussed below (Fig. 1, Additional file 1, and Additional file 2). A total of 183 organisms (113 bacteria, 10 archaea, and 60 eukaryotes) have been subjected to manual GEM reconstruction. We first discuss the current status of GEMs built for model organisms that are scientifically, industrially, and/or medically important, and then cover computational resources for GEM reconstruction.

### High-quality GEMs for model organisms

The GEMs for model organisms that have high scientific, industrial, and/or medical values have been updated several times since their initial reconstruction as more relevant biological information became available over the years. GEMs are often updated by adopting up-to-date experimental information on GPR associations and cell growth under various conditions (such as in gene knockouts or when different carbon sources are used), and by resolving issues such as incorrect GPR associations and different database identifiers for the same metabolite. The resulting GEMs serve as an excellent knowledgebase for studying the metabolism of the target organisms and are capable of predicting the organism’s various biological capabilities. As a result, high-quality GEMs of several model organisms reveal the history, rationale behind, and future directions of GEM development. Furthermore, they serve as good reference models for developing GEMs of other related organisms.

#### Bacteria

##### *Escherichia coli*

Being a model organism for bacterial genetics, the Gram-negative bacterium *Escherichia coli* has been subjected to genome-scale metabolic reconstruction campaigns for almost two decades. The first *E. coli* GEM, iJE660 [8], was reported in 2000 soon after the first release of the genome sequence of *E. coli* K-12 MG1655 [17]. The iJE660 model has subsequently been updated in terms of the coverage of GPR associations and prediction capacities, officially at least four times [18–21]. The most recent version, iML1515, contains information on 1515 open reading frames, twice the number of open reading frames incorporated in the original iJE660 model. The iML1515 model shows 93.4% accuracy for gene essentiality simulation under minimal media containing 16 different carbon sources such as glucose, xylose, and acetic acid. Importantly, iML1515 was tailored in various ways to extract the most relevant knowledge from a large volume of biological data. For example, iML1515-ROS has additional reactions associated with the generation of reactive oxygen species (ROS) and is useful for antibiotics design; iML976, a subset of iML1515, only contains information on metabolic genes that are shared by over 1000 *E. coli* strains and provides understanding of the core and accessory metabolic capacities of *E. coli* strains, especially clinical ones; and context-specific GEMs, by using proteome data from cells grown under specific conditions, reduce the false-positive predictions [21]. As a ‘model’ GEM, iML1515 shows how a GEM can accurately predict cellular metabolic and physiological states, and is expected to evolve further as new data become available.

##### Bacillus subtilis

*Bacillus subtilis* is a representative Gram-positive bacterium that has value to industrial biotechnology in the production of various enzymes and proteins [22, 23]. GEMs reconstructed for *B. subtilis* include iYO844 [24], a GEM by Goelzer et al. [25], iBsu1103 [26], iBsu1103V2 [27], iBsu1147 [28], and iBsu1144 [29]. The latest version, iBsu1144, built on the basis of the re-annotated genome information [30], was developed by incorporating thermodynamic information on the standard molar Gibbs free energy change for each reaction, in order to improve the accuracy and consistency of the reversibility of intracellular reactions. In one application study, iBsu1144 was employed to identify the effects of oxygen transfer rates (i.e., low, medium, and high oxygen transfer rates) on the production of serine alkaline protease and recombinant proteins using *B. subtilis* in silico. The *B. subtilis* GEMs will serve as important reference models for other Gram-positive bacteria.

##### *Mycobacterium tuberculosis*

In the fight against microbial pathogens, understanding their condition-specific metabolism (e.g., metabolism at a specific lifecycle point) at a systems level is very important for the identification of effective drug targets [31]. *Mycobacterium tuberculosis*, a bacterial pathogen that causes tuberculosis in humans, is one of the microbial pathogens that have been studied the most over the past 10 years using GEMs [32–38]. The most recent version of the GEM iEK1101 of *M. tuberculosis*, H37Rv, was developed by standardizing and combining biological information from previously released GEMs [38]. Upon reconstruction, iEK1101 was used to provide understanding of this pathogen’s metabolic status under an in vivo hypoxic condition, which replicates a pathogenic state, and also in an in vitro drug-testing condition. Comparing the predicted metabolic flux distributions in the two conditions allowed evaluation of the pathogen’s metabolic responses to antibiotic pressures [38]. Besides developing independent GEMs, a GEM of *M. tuberculosis* was integrated with a GEM of human alveolar macrophages to study host–pathogen interactions [39]. Development of systematic drug-targeting methods using GEMs of *M. tuberculosis* continues to be an active research area.

#### Archaea

##### Methanosarcina acetivorans

GEM reconstruction studies have mainly focused on prokaryotes and eukaryotes, but GEMs have also been built for a few archaea, such as *Methanosarcina acetivorans*, a methanogenic archaeal species which lives in a marine environment [40–43]. GEMs of *M. acetivorans* contain information on the methanogenesis pathway, the most representative metabolism of methanogens [44]. The iMAC868 model, the latest version of a *M. acetivorans* GEM [42], was established by integrating two previous models, iVS941 [40] and iMB745 [41]. iMAC868 was curated to represent a thermodynamically feasible methanogenesis reversal pathway that co-utilizes methane and bicarbonate, among other updates made in the GEM [42]. Another recent GEM of *M. acetivorans*, iST807 [43], was also updated on the basis of the previous version, iMB745 [41], in order to consider the effects of regulators on metabolic pathways in media containing different substrates. For example, in addition to the newly added metabolic pathways, tRNA-charging was explicitly incorporated into iST807, thereby allowing characterization of the effects of the differential expression of tRNA genes on metabolic fluxes. The GEMs for archaea will serve as a useful resource for metabolic studies on unusual characteristics of archaea in a wide range of habitats, including extreme environments and the human gut.

#### Eukarya

##### *Saccharomyces cerevisiae*

As the most representative eukaryotic microorganism, *S. cerevisiae* was the first eukaryotic organism to have its genome sequenced [45]. In addition, it was the first eukaryote for which a GEM was reconstructed [9]. Since the first *S. cerevisiae* GEM was released in 2003 [9], the GEMs for this microorganism have been updated by several different research groups [46–51]. However, the resulting different versions of *S. cerevisiae* GEMs were found to have inconsistent annotations, which hampered their comparison and further GEM upgrades. To address this inconsistency problem, a consensus metabolic network, Yeast 1, was reconstructed through an international collaborative effort [52]. Although Yeast 1 was a comprehensive metabolic network, it could not be simulated for flux predictions; constraint-based simulation became possible with a later version, Yeast 4 [53]. Yeast 1 has been upgraded to the latest version, Yeast 7, in the past few years by incorporating new biological information and by correcting critical modeling errors, such as the removal of thermodynamically infeasible reactions [53–56]. Very recently, Yeast 7.Fe [57] was extended from Yeast 7.6 [56] by including additional information on iron metabolism, which had not been properly considered in the previous GEMs. The Yeast 7.Fe now allows estimation of the optimal turnover rate of iron cofactors and more rigorous examination of metabolism. The GEMs of *S. cerevisiae* will continue to serve as reference models for eukaryotic microorganisms.

##### *Chlamydomonas reinhardtii*

The green microalgae *Chlamydomonas reinhardtii* has served as a model organism in studies of photosynthesis, phototaxis, cell motility, and bioenergy production [58, 59]. Owing to its biological importance, there has been much interest in reconstructing a GEM for *C. reinhardtii*. A total of six GEMs have been developed for *C. reinhardtii* to examine microalgal behaviors at the systems level, including iAM303 [60], iRC1080 [61], AlgaGEM [62], iCre1355 [63], a GEM by Winck et al. [64], and a GEM by Mora Salguero et al. [65]. The latest version by Mora Salguero et al. [65] allows dynamic simulations by including kinetic information on the effects of acetate as a nutrient on the growth rate at varying CO_2_ levels. Overall, the dynamic simulation of the *C. reinhardtii* GEM was able to predict cellular responses to the environmental changes accurately. GEMs for a greater number of microalgal species have been reconstructed for applications in biotechnology, including the production of lipids and secondary metabolites [66].

##### *Caenorhabditis elegans*

The nematode *Caenorhabditis elegans* has been employed as an established eukaryotic model organism in various studies, including work on aging [67], molecular and developmental biology [68], and nutrition [69]. The biological importance of *C. elegans* has led to multiple GEM reconstructions, including iCEL1273 [70], ElegCyc [71], and CeCon [72]. In 2017, the WormJam Community was founded to develop a consensus GEM of *C. elegans* by merging and reconciling the existing GEMs that had been developed by different research groups [73]. The three different *C. elegans* GEMs were merged to give a draft consensus GEM, and manual curation of the GEM was conducted to incorporate additional metabolic characteristics of *C. elegans*. The manual curation process, which was based on biological insights and over 40 metabolomics studies, led to the correction of errors in multiple metabolic pathways (i.e., glycogen metabolism, sphingolipid metabolism, and the biosynthesis and degradation of fatty acids, such as branched chain fatty acids) and the addition of new metabolic pathways (i.e., biosynthesis of maradolipids and ascaroside). The WormJam consensus GEM, the most accurate *C. elegans* GEM to date, now provides better biological insights into *C. elegans* physiology.

##### *Arabidopsis thaliana*

*Arabidopsis thaliana*, a model organism for plants, has also been an attractive target for extensive metabolic reconstruction studies, resulting in the development of four different GEMs: a GEM by Poolman et al. [74], AraGEM [11], a GEM by Mintz-Oron et al. [75], and a GEM by Cheung et al. [76]. Among these four GEMs, the latest version by Cheung et al. [76] was reconstructed to predict more accurate fluxes in the heterotrophic metabolism of *A. thaliana*, in particular by considering the transport costs associated with nutrient uptake and protein translocation between organelles and the maintenance costs for ATP and NADPH. By including information on these energy costs in the model, the metabolic flux distributions calculated using the updated GEM became more consistent with those obtained by ^13^C-metabolic flux analysis. Despite greater biological complexity, plant GEMs are beginning to be used more extensively to understand the metabolism of plants [77].

##### *Homo sapiens*

The availability of human GEMs has contributed to a better understanding of the biological mechanisms behind various diseases and to the design of appropriate disease treatments. Since the release of Recon 1, the first generic human GEM in 2007 [10], the Recon series has gone through several important updates, including the incorporation of additional biological information and the correction of various modeling errors [78–81]. Recon 2 M.2 is the version in which a framework for gene-transcript-protein-reaction associations (GeTPRA) was deployed to generate metabolic reactions by considering the effects of alternative splicing of metabolic genes (i.e., both principal and non-principal transcripts) [80]. Recon3D is the latest version and contains the most extensive human GPR associations and structural information on metabolites and enzymes. Recon3D can be used as a resource for many biomedical applications, including the characterization of disease-associated mutations and metabolic responses to drugs [81].

The Human Metabolic Reaction (HMR) series [82] is another generic human GEM series, which contains information on subcellular localization and tissue-specific gene expression, both mainly from the Human Protein Atlas database [83, 84]. In comparison with the Recon series, the HMR series has more comprehensive information on fatty acid metabolism that has been manually curated. The HMR series led to the generation of several cell-type-specific GEMs, such as *iAdipocytes1809* [82], *iHepatocytes2322* [85], and *iMyocyte2419* [86], which were used to study obesity, non-alcoholic fatty liver disease (NAFLD), and diabetes, respectively. Both the Recon and the HMR series will serve as useful resources for various biomedical studies, as discussed below.

### Computational resources for automatic GEM reconstruction

Manual reconstruction of GEMs is a time-consuming procedure [3], in which a large number of GPR associations and many other sources of data and information must be considered. To address this challenge, several software programs for automatic GEM reconstruction have been developed (Table 1). A significant part of the GEM reconstruction procedure has now been automated, including, but not limited to, the annotation of genome sequence, the generation of a set of GPR associations unique to a target organism, the prediction of reaction reversibility on the basis of thermodynamics, and enzyme localization. Three independent studies involving the high-throughput generation of GEMs, namely Path2Models [13], AGORA [14], and CarveMe [15], led to the reconstruction of more than 6000 GEMs (Table 2). CarveMe led to the generation of the greatest number of GEMs, especially for bacteria, followed by Path2Models and AGORA (Fig. 1). CarveMe is a computational pipeline that automatically converts a manually curated reaction dataset (known as the ‘universal model’) into a target-organism-specific GEM. CarveMe was used to reconstruct 5587 bacterial GEMs, which corresponds to 91.8% of the bacterial GEMs reconstructed (Fig. 1). Path2Models, the first large-scale GEM reconstruction project, allowed reconstruction of GEMs for 2606 organisms, which are accessible at the BioModels database [98]. GEMs developed through Path2Models cover 20.1, 98.4, and 88.8% of all the GEMs reconstructed for bacteria, archaea, and eukaryotes, respectively (Fig. 1). AGORA models include the GEMs of 773 members of the human gut microbiota, which were prepared in a semi-automatic manner using the online GEM reconstruction platforms Model SEED [93] and KBase [104]. As of February 2019, 818 AGORA models are accessible at the Virtual Metabolic Human database [103]. Both manually curated high-quality GEMs and automatically reconstructed GEMs are now available at several databases, as summarized in Table 2.

**Table 1:** Software programs for the reconstruction of GEMs

Tool	Language	Graphical user interface (GUI) available?	Source database for metabolic reactions	Use as a reference model?	Gap-filling	Eukaryote modeling	Simulation ready	Reference
AuReMe	Python	No	KEGG, BiGG Models, MetaCyc	No	Yes	Yes	Yes	[87]
AutoKEGGRec	Matlab	No	KEGG	No	No	No	No	[88]
CarveMe	Python	No	BiGG Models	Yes (universal model)	Yes	No	Yes	[15]
CoReCo	Python	No	KEGG	No	Yes	Yes	Yes	[89]
FAME	Python	Yes	KEGG	Yes	No	No	Yes	[90]
*merlin*	Java	Yes	KEGG, MetaCyc, UniProtKB, TCDB	No	No	Yes	No	[91]
MetaDraft	Python	Yes	BiGG Models	Yes	No	No	Yes	[92]
Model SEED	Web	Yes	In-house reaction database	Yes	Yes	Yes (only plants)	Yes	[93]
Pathway Tools	Python, Lisp	Yes	Pathway/Genome Database (PGDB), MetaCyc	No	Yes	Yes	Yes	[94]
RAVEN 2.0	Matlab	No	KEGG, MetaCyc	Yes	Yes	Yes	Yes	[95]
SuBliMinal Toolbox	Java	No	KEGG, MetaCyc	No	No	No	Yes	[96]

**Table 2:** Representative GEM databases

Database	Available GEMs	URL	Reference(s)
BiGG Models	84 high-quality GEMs that were manually reconstructed	http://bigg.ucsd.edu	[97]
BioModels	6753 patient-specific GEMs using TCGA datasets, > 2600 GEMs from the Path2Models project, and 641 manually curated small-scale metabolic models from the literature	http://www.ebi.ac.uk/biomodels	[98]
Human Metabolic Atlas (HMA)	Two generic and > 100 context-specific human GEMs, five human gut microbiota species GEMs, and a mouse GEM	http://www.metabolicatlas.org	[99]
MEMOSys 2.0	20 publicly available GEMs in a standardized format	http://memosys.i-med.ac.at (unstable)	[100]
MetaNetX	217 GEMs collected from GEM-relevant databases (e.g., BiGG Models and Model SEED) in a standardized format	http://www.metanetx.org	[101]
Model SEED	39 plant GEMs reconstructed using PlantSEED	http://modelseed.org	[93, 102]
Virtual Metabolic Human (VMH)	818 GEMs for gut microbes (AGORA models) and Recon3D	http://www.vmh.life	[103]

As these tools and strategies for GEM reconstruction are advancing rapidly, an increasing number of GEMs for many different organisms, including those of research interest, have become available. Nevertheless, challenges remain with the automatic GEM reconstruction. The most urgent challenges are to evaluate the quality of an automatically generated draft GEM and to automate the refinement procedure [105]. A draft GEM usually includes inaccurate reactions and GPR associations; for example, the biomass generation reaction is not tailored for a target organism, and reactions are often incorrectly constrained (e.g., there are no constraints or incorrect reaction reversibility in the draft GEM). Solutions exist to meet this challenge. The quality of the draft GEMs can be evaluated by using a set of task functions that are relevant to a target organism; this feature is now addressed by the software program memote (which stands for ‘*me*tabolic *mo*del *te*sts’) [106], but in an organism-independent manner. Some established algorithms for refining draft GEMs, such as gap-filling [107, 108] and the integration of experimental data (such as those from cultivation experiments under various conditions) [5], can be integrated with the GEM reconstruction tools to allow (semi-)automatic refinement of the draft GEMs. For complex problems such as the formulation of an organism-specific biomass generation reaction, manual refinement assistance could be provided in the GEM reconstruction tool, for example, by providing the biomass generation equations of biologically related organisms. In the future, it is expected that this step will also be automated, for example, by automatically extracting information on a target organism-specific biomass composition and even GPR associations from the literature using text mining techniques.

## Applications of GEMs

GEMs of various organisms have been widely employed in scientific discovery as well as in various industrial and medical applications [7, 109, 110]. Importantly, the development of omics data integration methods for GEMs resulted in the expansion of the application scope of GEMs [111] by tailoring a GEM according to specific conditions of interest. Relevant omics data integration algorithms [5, 112, 113] include GIMME [114], iMAT [115], MBA [116], INIT [117], mCADRE [118], tINIT [119], CORDA [120], and TIMBR [121]. The integration of omics data with GEMs is particularly important for modeling multicellular organisms, such as human and plants, because the generic GEMs that are available for these organisms need to be transformed into context-specific GEMs. Generic GEMs do not address condition-specific metabolism because they have information on all metabolic genes regardless of their expression levels in a specific tissue or cell type. Relevant studies involving context-specific GEMs include the prediction of condition-specific (e.g., specific to life cycle stage or cultivation environment) drug targets in pathogens, the prediction of host–pathogen metabolic interactions, and the characterization of the reprogrammed metabolism of liver cancer stem cells (LCSCs) and the endothelium of sepsis patients, which are all discussed below. Further details of various omics data integration methods have been thoroughly discussed elsewhere [5, 112, 113].

### Production of chemicals and materials

**Fig. 2:**
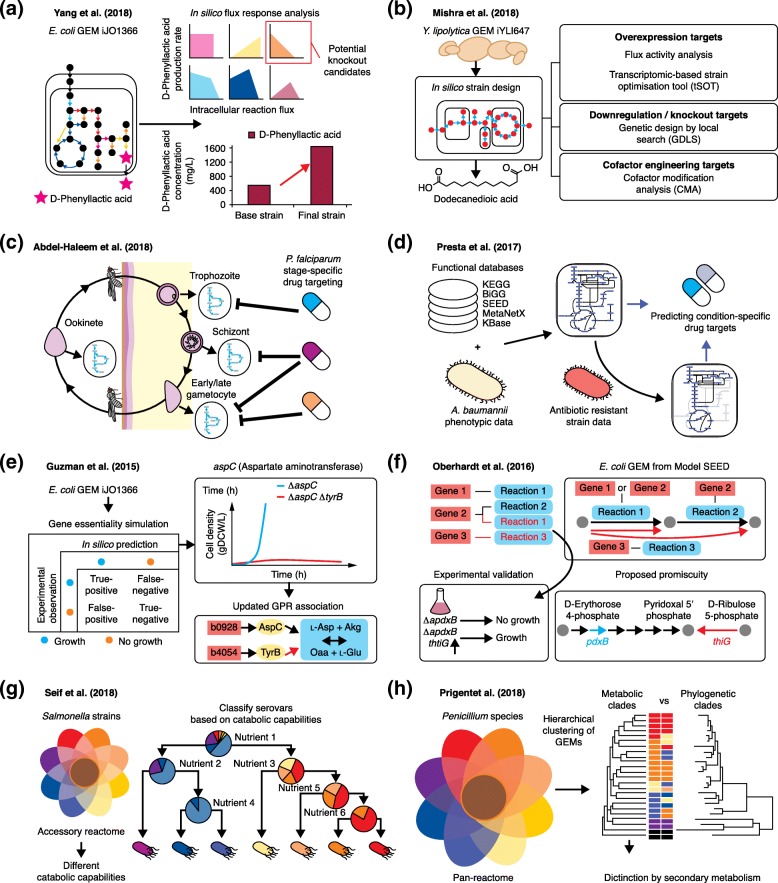
Applications of GEMs for the production of chemicals and materials, drug targeting in pathogens, the prediction of enzyme functions, and pan-reactome analysis. **a.** Flux-response analysis using the *Escherichia coli* GEM iJO1366 [20] has been used to identify gene manipulation targets for the enhanced production of a monomer of an aromatic polymer d-phenyllactic acid in *E. coli* [122]. The final strain has two additional genes (*tyrB* and *aspC*) knocked out from an *E. coli* base strain XB201T that expresses AroG^fbr^, PheA^fbr^, and FldH. The final strain produced 1.62 g/L of d-phenyllactic acid, much more than the 0.55 g/L produced by the base strain. **b** Reconstruction of the *Yarrowia lipolytica* GEM iYLI647 and its application for the prediction of reaction engineering targets using four different in silico strain-design strategies [123]. **c** Identification of stage-specific antimalarial drug targets for *Plasmodium falciparum* using stage-specific GEMs that represent five different life cycle stages [124]. **d** Reconstruction of a GEM for *Acinetobacter baumannii* using several databases and its application for the prediction of condition-specific drug targets to combat antibiotic-resistant *A. baumannii* [125]. **e** Discovery of new isozyme functions for genes that have been shown to be nonessential in experiments but that were predicted to be essential in a gene essentiality simulation of the *E. coli* iJO1366 GEM (i.e., false-negative prediction for the *aspC* gene) [126]. It was found that tyrosine aminotransferase, which is encoded by *tyrB* (*red line*), can compensate for the loss of aspartate aminotransferase, encoded by *aspC*, which catalyzes the conversion of l-aspartate (l-Asp) and α-ketoglutarate (Akg) to oxaloacetate (Oaa) and l-glutamate (l-Glu). *gDCW/L* grams dry *cell* weight per liter. **f** The PROmiscuity PrEdictoR (PROPER) method identifies promiscuous enzymes at a genome-scale in a target organism [127]. Promiscuous functions for all of the genes in the target organism (*E. coli*) were predicted using the PROPER method and an *E. coli* GEM from the Model SEED, which identified 98 alternative routes for the biosynthesis of various metabolites. For example, the product of the *thiG* gene in *E. coli* was newly found to biosynthesize pyridoxal 5′-phosphate, which is also known to be biosynthesized by the product of the *pdxB* gene. **g** Analysis of the pan-reactome and accessory reactome of 410 *Salmonella* strains spanning 64 serovars using their respective GEMs [128]. Simulation of the GEMs under various nutrient conditions revealed the different catabolic capabilities of the different strains as well as their preferred growth environments. **h** Analysis of the pan-reactome and accessory reactome of 24 *Penicillium* species by using their respective GEMs [129]. Hierarchical clustering of the 24 GEMs revealed additional insights into the biosynthetic pathways of secondary metabolites, which successfully differentiated the metabolic clades

GEMs have long been used to predict targets for effective gene manipulation (by knockout or through the up- or downregulation of gene expression, for example) for the enhanced microbial production of chemicals and materials. Notably, GEMs have been applied to redesign aspects of the metabolism of both bacteria and eukaryotes in order to produce an increasing number of chemicals and materials. A recent example is the enhanced production of aromatic polymers involving d-phenyllactic acid as a monomer (e.g., poly(3-hydroxybutyrate-co-d-phenyllactate)) using metabolically engineered *E. coli* strains (Fig. 2a) [122]. Direct production of d-phenyllactic acid from glucose was first attempted by implementing flux response analysis of the *E. coli* GEM iJO1366 [20] to examine the effects of engineering central and aromatic amino acid biosynthetic reactions on d-phenyllactic acid production. Additional knockouts of *tyrB* and *aspC* genes in an engineered *E. coli* base strain (XB201T) producing 0.55 g/L of d-phenyllactic acid successfully increased d-phenyllactic acid production to 1.62 g/L. Fed-batch fermentation of the final strain produced 13.9 g/L of poly(61.9 mol% 3-hydroxybutyrate-co-38.1 mol% d-phenyllactate).

In another example involving *Yarrowia lipolytica*, an eukaryotic microorganism known to accumulate large amounts of lipids [130], its GEM was used to improve the production of dodecanedioic acid [123] (Fig. 2b). First, a GEM of *Y. lipolytica*, iYLI647, was newly reconstructed and employed to find target reactions that can lead to the enhanced production of dodecanedioic acid [123]. For this, several in silico strain design methods were implemented using iYLI647, including (1) flux activity analysis (a method examining the effects of changes in individual reaction fluxes on a target chemical production rate) [131] and the transcriptomics-based strain optimization tool (tSOT) [132], both of which identify overexpression targets; (2) genetic design by local search (GDLS) [133], which is used to identify knockout targets; and (3) cofactor modification analysis (CMA) [134], which identifies cofactor modification targets. Application of algorithms such as these to redesign a microbial strain’s metabolism allows the identification of more robust gene manipulation targets, and is becoming an essential practice in metabolic engineering.

### Drug targeting in pathogens

Another important application of GEMs is to predict the viability of an organism under a given condition. This simulation approach has been utilized to suggest metabolic drug targets whose inhibition can effectively kill a pathogen. The GEM of a target pathogen can be used to predict essential genes (or reactions) [32, 135] and essential metabolites [136, 137], each of which can lead to a different drug discovery strategy. A recent study using GEMs suggested drug targets in *Plasmodium falciparum* that are specific to the life cycle stage of the malaria-causing pathogen [124]. *P. falciparum* goes through a complex life cycle to reproduce itself [138]. Because each life cycle stage has a different metabolic network structure, it is likely that different drug targets can be found for each stage. Thus, the stage-specific GEMs of *P. falciparum* were reconstructed [124]. Integration of a generic GEM with stage-specific transcriptome and physiology data, such as stage-specific growth rates and metabolite secretion rates, led to five stage-specific models that represent the trophozoite, schizont, early gametocyte, late gametocyte, and ookinete (Fig. 2c). Gene essentiality analysis of the stage-specific GEMs showed 71.2% accuracy in comparison with experimentally characterized drug targets (42 out of 59 drug targets). The prediction outcome indicates that the quality of the *P. falciparum* GEM needs to be improved further. In addition, new drug targets beyond these 59 targets need to be identified, especially novel targets that are effective in the proliferative and late gametocyte stages. Life-cycle-stage-specific modeling and simulation approaches such as this will be important for drug targeting in other pathogens that exhibit different life stages, but this approach requires the acquisition of stage-specific omics data.

Condition-specific drug targeting using GEM has also been conducted for *Acinetobacter baumannii* [125], which is one of the six ESKAPE pathogens (*Enterococcus faecium*, *Staphylococcus aureus*, *Klebsiella pneumoniae*, *A. baumannii*, *Pseudomonas aeruginosa*, and *Enterobacter* spp.) associated with antimicrobial resistance [139]. Specifically, an updated version of the *A. baumannii* GEM, iLP844, was reconstructed and transformed into condition-specific GEMs by integration with transcriptome data. The transcriptome data were obtained from cells treated and untreated with colistin, one of the last-resort antibiotics against multi-drug-resistant pathogens [125]. Condition-specific drug targets were obtained by predicting genes that are exclusively essential in colistin-treated cells, and not homologous to any human genes, so that possible side-effects in the human body are avoided (Fig. 2d). It should be noted that similar approaches have also been applied to predict drug targets for diseased human cells such as cancers [119, 140]. Once a condition-specific GEM is built, drug targets can be predicted relatively easily. More demanding challenges are to validate the targets experimentally and to identify drugs that can effectively inhibit the predicted drug targets.

### Prediction of enzyme functions

Rigorous analysis of the simulation results from a GEM also allows the identification of previously unidentified reactions or enzyme functions. In this context, two representative studies demonstrate how GEMs can be used to unveil additional functions of an enzyme. One study focused on a set of genes that were shown in experiments to be nonessential, but that were predicted to be essential in a gene essentiality simulation of the *E. coli* GEM iJO1366 (i.e., there was a false-negative prediction of cell growth; Fig. 2e) [126]. Such false-negative predictions were thought to be caused by the presence of previously unidentified reactions that made a nonessential gene essential upon its knockout in silico. Among the ‘false-negative’ genes, *aspC*, *argD*, and *gltA* were selected for experimental validation because sequence homology analysis identified high-confidence candidate isozymes. Knockout of the genes encoding the potential isozymes revealed that tyrosine aminotransferase, which is encoded by *tyrB*, can compensate for the loss of aspartate aminotransferase, which is encoded by *aspC* (Fig. 2e). The same knockout approach also identified potential isozymes that could serve as alternative reaction enzymes for those encoded by *argD* and *gltA*.

In another study, a new method called PROmiscuity PrEdictoR (PROPER) [127] was developed to identify promiscuous enzymes in a target organism at the genome scale. For the implementation of PROPER, gene-similarity trees were built for all of the genes in *E. coli* using Position-Specific Iterated (PSI) BLAST, which show their homologous genes from the SEED database. The gene-similarity trees were used to generate a matrix that presents the primary and potential promiscuous functions (i.e., metabolic reactions) of *E. coli* enzymes encoded by the corresponding genes. Finally, ‘replacer’ genes were identified in the matrix, which have a potential promiscuous function that is identical to the primary function of another conditionally essential gene (‘target’ gene) in *E. coli*. A potential promiscuous function of a replacer gene can be validated if expression of this gene can prevent cell death upon knockout of the target gene. Among the target–replacer gene pairs predicted using the PROPER method and an *E. coli* GEM from Model SEED [93], the *pdxB*–*thiG* gene pair was experimentally validated (Fig. 2f). The *pdxB* gene is a conditionally essential gene involved in the biosynthesis of pyridoxal 5′-phosphate in *E. coli*, and served as a target gene in this study. The *thiG* gene, a replacer gene in this study, encodes 1-deoxy-d-xylulose 5-phosphate:thiol sulfurtransferase, an enzyme in the thiazole biosynthetic pathway, which was shown to biosynthesize pyridoxal 5′-phosphate without involving the enzyme encoded by *pdxB*.

These two studies have demonstrated that a high-quality GEM of a target organism allows the prediction of new enzyme functions and enzyme promiscuity, which is extremely useful because not all enzyme functions are experimentally validated.

### Pan-reactome analysis

Computational resources for high-throughput GEM reconstruction are now allowing the metabolic analysis of multiple organisms, including multiple strains of a single species [141, 142] or multiple species of a single genus [128, 129]. Analysis of a pan-reactome, an entire set of reactions, of biologically related organisms using GEMs provides a better understanding of the metabolic traits and lifestyles of these organisms. This concept was applied to study the metabolic traits of 410 *Salmonella* strains, spanning 64 serovars, by reconstructing a GEM for each strain [128]. The constructed pan-reactome revealed that the metabolic differences among the strains come from the accessory reactome, a set of reactions that are present in only some strains. These reactions were largely involved in alternative carbon metabolism and in cell wall or membrane metabolism. In particular, the strains could be distinguished on the basis of their different catabolic capabilities by analyzing their growth under various nutrient conditions in silico (Fig. 2g). Further investigation of serovar-specific catabolic capabilities helped to reveal the growth environments that are preferred by the *Salmonella* serovars and provided information about their evolution. Automatic GEM reconstruction tools have definitely contributed to pan-reactome analysis, and will continue to be applied to various groups of biologically related organisms of high scientific, industrial, and/or medical importance.

Along the same lines, a pan-reactome analysis was conducted to provide information on the metabolic features of 24 *Penicillium* species that are well-known for the production of secondary metabolites [129]. Analysis of the 24 reconstructed GEMs revealed that most of the reactions involved in primary metabolism were conserved among these species. Subsequent hierarchical clustering of the 24 GEMs showed that the biosynthetic pathways for secondary metabolites were the most distinctive pathways in differentiating the metabolic clades, and that these pathways contributed to the genomic diversity of the 24 *Penicillium* species (Fig. 2h). Comparison of the metabolic clades with the phylogenetically classified clades, which were based on entire protein sequences for each species, demonstrated that stratifying species solely by using the phylogenetic tree could not fully explain the metabolic differences among the species. These representative studies demonstrate that the use of GEMs can bring about additional biological insights into a group of biologically related organisms. In the near future, an automated GEM refinement procedure using experimental data will much improve the quality of pan-reactome analysis, which at present is mainly conducted using draft GEMs.

### Modeling interactions among multiple cells or organisms

**Fig. 3:**
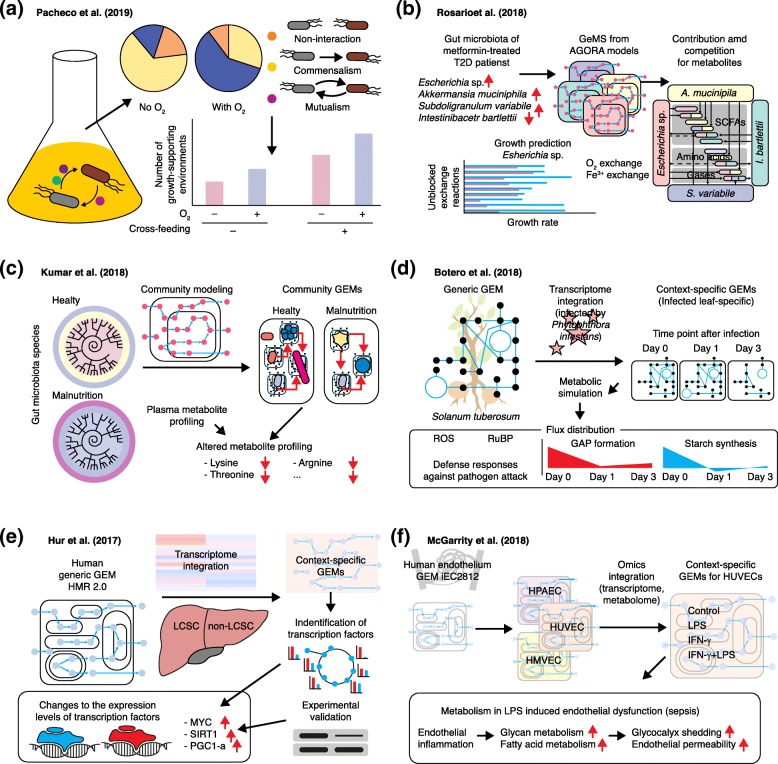
Applications of GEMs for interspecies metabolic interactions and understanding human diseases. **a** GEM-based simulation of the effects of costless metabolites (i.e., metabolites that have no effects on the producing organism’s growth rate) secreted by at least one of two paired microorganisms on their growth under anaerobic and aerobic conditions [144]. The number of growth-supporting environments was increased as a result of cross-feeding. **b** Prediction of the metabolites (e.g., short-chain fatty acids [SCFAs]) required or produced by four representative gut microbiota species, *Escherichia* sp., *Akkermansia muciniphila*, *Subdoligranulum variabile*, and *Intestinibacter bartlettii*, which are known to be affected by the type 2 diabetes (T2D) drug metformin [146]. **c** Prediction of the metabolites produced by gut microbiota species from malnourished children using community GEMs that describe the metabolism of multiple gut microbiota species [147]. The prediction results were consistent with the children’s plasma metabolite profiles. **d** Prediction of the suppressed photosynthesis of a potato plant (*Solanum tuberosum*) upon infection by the plant pathogen *Phytophthora infestans*, which triggers the plant’s defense responses against pathogen attack through oxygenation of ribulose1,5-bisphosphate (RuBP) and subsequently increases in the intracellular levels of reactive oxygen species (ROS) [148]. Formation of glyceraldehyde-3-phosphate (GAP) and starch were also decreased as a result of the infection. **e** Identification of metabolic differences between liver cancer stem cells (LCSCs) and non-LCSCs, and of the transcription factors responsible for the metabolic changes, by using GEMs integrated with transcriptome data [149]. **f** Characterization of the reprogrammed metabolism of the endothelium cells of sepsis patients using a human endothelium GEM, iEC2812 [150]. Context-specific GEMs were created using transcriptome and metabolome data obtained from human umbilical vein endothelial cells (HUVECs) treated with lipopolysaccharide (LPS) and/or interferon-γ (IFN-γ). Simulation of the context-specific GEMs indicated that increased glycan and fatty acid metabolism led to increased glycocalyx shedding and endothelial permeability where there was endothelial inflammation. *HPAEC* human pulmonary artery endothelial cell, *HMVEC* human microvascular endothelial cell

The modeling of metabolic interactions among multiple cells or organisms is also an important application of GEMs. This approach has been used for various studies of intermicrobial interactions, including the cross-feeding of microorganisms (or the exchange of metabolites between microorganisms) [92, 143, 144] and the evolutionary trajectory of microbial communities [145]. A recent study using GEMs has revealed that the secretion of costless metabolites contributes to the better growth of other interacting microorganisms, and ultimately to a greater taxonomic diversity in nature (e.g., in a nutrient-poor environment) [144]. Costless metabolites were defined as those that do not negatively affect the producing organism’s fitness cost (i.e., growth rate) upon secretion [144]. The pairwise growth of the 24 microbial species examined in this study was simulated under various environmental conditions, involving different carbon sources and varying availability of oxygen, in order to examine the effects of the paired microorganisms’ cross-feeding on their growth (Fig. 3a). The number of media that allowed the growth of at least one of the two microorganisms substantially increased if the exchange of costless metabolites between the microorganisms was allowed in the simulation. Interestingly, more frequent bidirectional exchanges between the two microorganisms and a greater number of costless metabolites were observed under anaerobic conditions than under aerobic conditions. These carefully designed in silico simulations using GEMs allowed the identification of new biological insights into intermicrobial interactions at a scale that would be difficult to replicate experimentally.

In a study involving type 2 diabetes patients treated with the drug metformin [146] (Fig. 3b), the metabolism of four representative gut microbiota species, *Escherichia* sp., *Akkermansia muciniphila*, *Subdoligranulum variabile*, and *Intestinibacter bartlettii*, was examined using their respective GEMs. The GEMs were obtained from the AGORA models. Upon metformin treatment, *Escherichia* sp., *A. muciniphila*, and *S. variabile* are known to be enriched in the gut, while *I. bartlettii* is reported to decrease. In the simulation studies, contributing or competing bacteria were predicted through simulation of the GEMs for short-chain fatty acids (e.g., acetic and butyric acids), amino acids, and gases (e.g., H_2_, H_2_S, and CH_3_SH), all of which play important roles in both intermicrobial metabolic interactions and the regulation of human metabolism. For example, *Escherichia* sp. and *S. variabile* were predicted to contribute to the production of short-chain fatty acids under aerobic and anaerobic conditions. In addition, *Escherichia* sp. was shown to be least affected by the availability of intestinal nutrients. Covering a greater range of microorganisms and the metabolites exchanged among them will add more scientific value to GEM-based studies of a specific microbiota.

In this regard, another recent study also deserves attention. Kumar et al. [147] examined the production of metabolites by gut microbiota in children with malnutrition using GEMs for 58 representative gut microbiota species (Fig. 3c) [147]. The GEMs for 58 representative gut microbiota species were reconstructed using Model SEED and then used to examine metabolic differences (i.e., common and unique reactions) among these microorganisms. Community metabolic models (CMMs) were also reconstructed by integrating the GEMs of individual microbiota species according to the composition of the gut microbiota; each CMM represents the entire gut microbiota species of each child. Simulation of the CMMs revealed that the production of essential amino acids by the gut microbiota of the malnourished children was decreased, which was consistent with the children’s plasma metabolite profiles. The development of strategies for treating abnormal health conditions on the basis of findings from GEMs will be a major challenge for the near future.

The metabolic interaction between a host and a pathogen is another important type of interspecies interaction that can be studied using GEMs [151]. In one recent study, the effects of pathogen infection on the host plant’s photosynthetic capacity were examined using GEMs [148]. A generic GEM of the leaf of a potato plant (*Solanum tuberosum*) was first reconstructed, and three context-specific GEMs were subsequently created by incorporating transcriptome data from the cells of plants that were infected with *Phytophthora infestans*, a plant pathogen that causes late blight, at days 0, 1, and 2 after infection (Fig. 3d). The three context-specific GEMs were subsequently used alone to infer the metabolic interactions without using the pathogen’s GEM. Pathogen infection was predicted to affect Calvin cycle fluxes, and thus carbon fixation. In particular, at day 1 after infection, the carboxylase activity and oxygenase activities of ribulose-1,5-bisphosphate carboxylase/oxygenase (RuBisCO), the first enzyme committed to carbon fixation in the Calvin cycle, were predicted to be decreased and increased, respectively. Such changes are known to reduce photosynthesis, and subsequently to induce the production of ROS, which could be associated with a quick defense mechanism against pathogen attack. Moreover, the flux of glyceraldehyde 3-phosphate formation in the second part of the Calvin cycle, as well as starch biosynthesis flux (an indicator of plant health), was predicted to decrease drastically from day 0 to day 1, but to recover slightly at day 3. GEM-based studies of strategies to protect plants against pathogens by examining fluxes of Calvin cycle and other pathways, which indicate the health status of a plant, will be of interest.

Modeling interactions among multiple cells or organisms, especially microbiota, presents many technical challenges. First, the microbial species that constitute a specific microbiota are not fully elucidated in most, if not all, cases. This partly explains why the microbial communities covered by the studies described above were simplified by considering only representative microbial species. Thus, the use of GEMs will become more powerful when it becomes possible to identify all (or at least most) microbial species in a given community. For example, key microorganisms or metabolites in a specific microbiota can be suggested more systematically by examining metabolic interactions among more varied combinations of microorganisms from the microbiota. Here various modeling and simulation algorithms beyond FBA can be developed, depending on the objective of study and the scale of the metabolic modeling to be examined. Second, it is extremely difficult to measure metabolites that are exchanged by microbial species in vivo. Metabolome analyses using food, stool, and/or serum have been the most frequently practiced approaches for characterizing the metabolism of microbiota species, but still have limitations in revealing metabolite exchange by microbial species in vivo. This issue has led to active discussions on the need to identify and apply accurate condition-specific constraints for the GEMs of gut microbiota species and to conduct community-level manual curation of the GEM of each gut microbiota species [152, 153]. Finally, it is very important to inform experimental microbiologists of how the GEMs of microbiota are reconstructed, how omics data are used to improve the GEMs, and what the GEMs of microbiota species can be used for. Taken together, the development of experimental and computational techniques for the accurate measurement of metabolites in vivo and proper communication with experimental microbiologists will allow better understanding of microbe–microbe and host–microbe interactions. This will be important because the GEMs of microbiota species tend to present flux predictions that are often distinct from those of model organisms [152, 153].

### Understanding human diseases

Human diseases have also been studied using context-specific GEMs to elucidate metabolic malfunctions in cells that are under chronic or acute disease conditions, and to suggest effective therapeutic targets. Cancers, including cancers of the liver [140, 154–156], breast [157], prostate [158], lung [159], and colon-rectum [160], have been the most active target of context-specific GEMs. Chronic diseases, including NAFLD [161] and obesity [140], have also been examined using context-specific GEMs. In a study by Hur et al. [149], the metabolism of LCSCs that showed therapeutic resistance in hepatocellular carcinoma was investigated in comparison with non-LCSCs by building context-specific GEMs for both cell types using their transcriptome data [149] (Fig. 3e). Upon identifying reactions with fluxes that differed significantly between LCSCs and non-LCSCs, transcription factors that are known to be associated with those reactions were traced. As a result, MYC, a transcription factor that is important in cell proliferation, among other transcription factors, was found to be heavily involved in the changed metabolism of the LCSCs. This prediction was experimentally validated, providing insights into the reprogrammed metabolism of LCSCs. This GEM-based comparative analysis, along with the use of relevant omics data, is also applicable to explaining the reprogrammed metabolism of other types of cancer cells or other abnormal cells that represent disease conditions.

Other studies have used GEMs to predict altered intracellular metabolic flux distributions in acute diseases such as sepsis [150] and viral infection [162]. In one study, the reprogrammed metabolism of endothelium in sepsis patients was investigated (Fig. 3f) [150]. A human endothelium GEM, iEC2812, was reconstructed and integrated with transcriptome data to represent the metabolism of three endothelial subtypes: human pulmonary artery endothelial cell (HPAEC), human umbilical vein endothelial cell (HUVEC), and human microvascular endothelial cell (HMVEC). The network structures of the three context-specific GEMs were compared with one another in order to identify metabolic differences among the three endothelial subtypes, which occurred mainly in nucleotide metabolism. Furthermore, context-specific GEMs for HUVECs were reconstructed using transcriptome and metabolome data, which were obtained from lipopolysaccharide (LPS) and/or interferon-γ (IFN-γ)-treated HUVECs. The treatment of endothelial cells with LPS and IFN-γ triggers a cellular status similar to those seen under bacterial infection and during an immune response, respectively. These context-specific GEMs for the LPS- and IFN-γ-treated HUVECs predicted elevated fluxes through glycan and fatty acid metabolism, which were found to increase glycocalyx shedding and endothelial permeability in the sepsis patients.

Human diseases are associated with highly complex cues and cascading of signals, and thus, the use of GEM-based simulations alone can provide only limited insights into disease. In the future, a number of important studies need to be performed to provide a better understanding of human diseases and to help in designing proper therapies. First, in addition to a metabolic network, regulatory and/or signaling networks should also be considered to allow a more accurate computational description of a diseased cell. These different types of biological network are connected with one another in a highly complex manner. Thus, it will ultimately be necessary to integrate metabolic, gene regulatory, and signaling networks in modeling and simulation. This will require an innovative computational framework that allows simultaneous simulation of material flow (metabolic network) and information flow (gene regulatory and signaling networks). Second, it is increasingly recognized that a number of human diseases are significantly affected by patients’ lifestyles. Thus, it will be necessary to develop strategies to integrate human GEMs with a framework of precision medicine that involves not only patient-specific omics data but also personal lifestyle data, such as dietary habits and patterns of various physical activities.

## Conclusions

Having been established as one of the major modeling approaches for metabolic studies at systems level, the reconstruction and simulation of GEMs continue to be explored for an increasingly wider range of organisms and applications. Advances in the reconstruction and use of GEMs are largely attributed to the greater availability of biological data and information, and to the establishment of automatic GEM reconstruction tools. GEMs will continue to evolve by embracing a greater coverage of GPR associations, reconciling model inconsistencies, and developing novel mathematical modeling techniques that can be used in a high-throughput manner. GEMs will become more powerful by incorporating additional biochemical information that will provide explanations of cellular processes beyond metabolism [163]. Additional information that has been incorporated into GEMs successfully includes protein allocation [164–167], cellular macromolecular composition [168, 169], and protein structural information [21, 170–172]. Nevertheless, various biochemical properties, such as enzyme–substrate interactions, the structure of protein–protein complexes, and post-translational modification, still need to be considered further. It is expected that GEMs will find increasing applications in studying interactions among a greater number of cell types. These will include, for example, interactions among microorganisms in a given microbiota under spatiotemporally varying conditions, metabolic interactions between human (or animal or plant) cells and microbiota, and interactions among multiple human cells, to name a few. Although technical challenges remain to be overcome, GEMs will be applied to study an expanding and increasingly complex range of problems.

## Additional files

Additional file 1: **Figure S1.** A phylogenetic tree at the species level of all of the GEMs reconstructed to date. (PDF 9989 kb)

Additional file 2: **Table S1.** A full list of organisms subjected to the GEM reconstruction and used in the preparation of phylogenetic trees in Fig. 1 and Additional file 1. (XLSX 305 kb)

## Abbreviations

CMM: Community metabolic model
FBA: Flux balance analysis
GEM: Genome-scale metabolic model
GPR: Gene-protein-reaction
HMR: Human metabolic reaction
HUVEC: Human umbilical vein endothelial cell
IFN-γ: Interferon-γ
LCSC: Liver cancer stem cell
LPS: Lipopolysaccharide
NAFLD: Non-alcoholic fatty liver disease
PROPER: PROmiscuity PrEdictoR
ROS: Reactive oxygen species
