# Supplementary material for: Current status and applications of genome-scale metabolic models
Source: Genome Biol. 2019 Jun 13;20:121. doi: 10.1186/s13059-019-1730-3 (PMC6567666; doi:10.1186/s13059-019-1730-3)
Supplement: Supplementary file 1 — Figure S1. A phylogenetic tree at the species level of all of the GEMs reconstructed to date. (PDF 9989 kb) [file 13059_2019_1730_MOESM1_ESM.pdf]

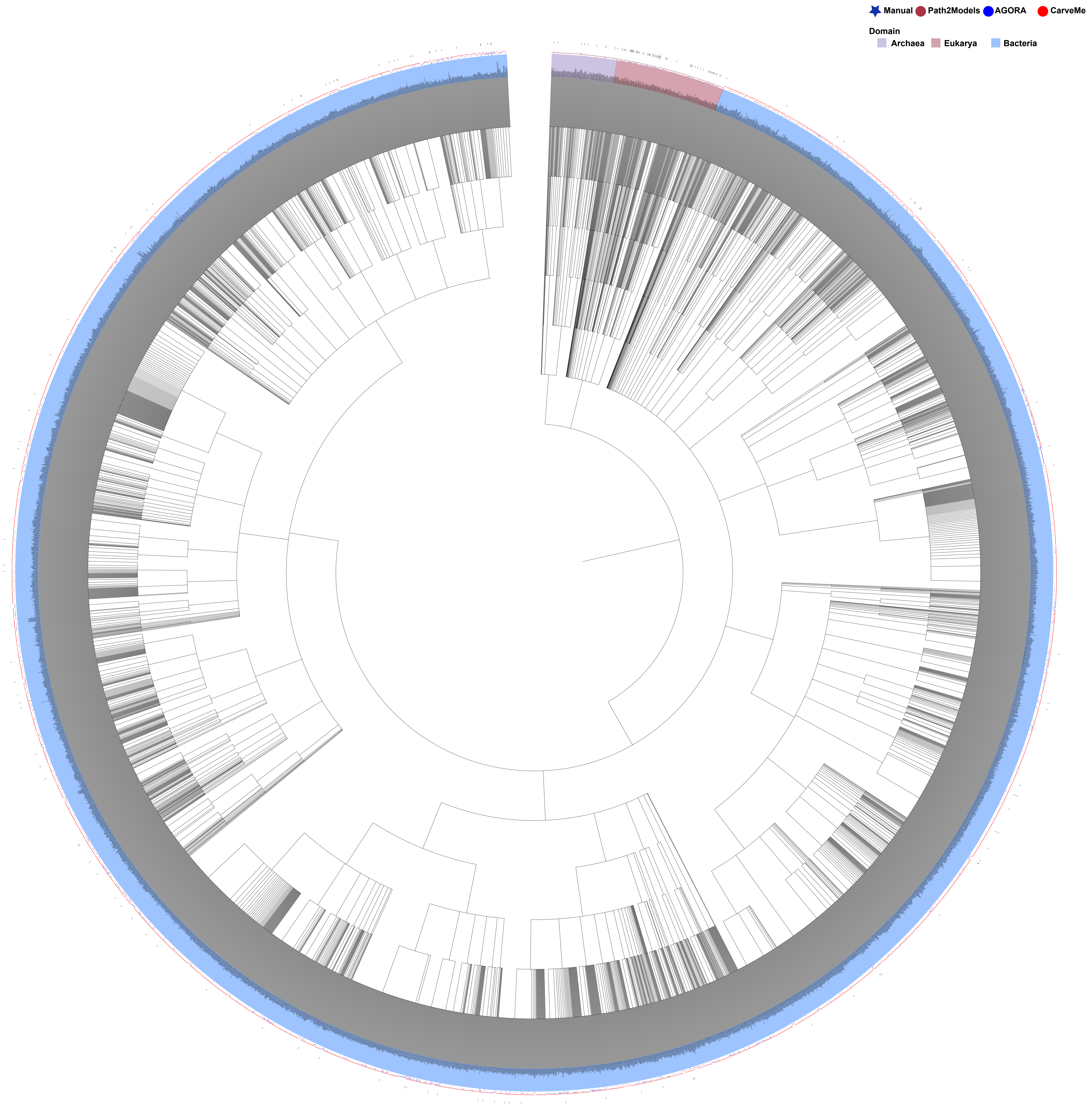

Fig. S1 A phylogenetic tree at the species level of all of the GEMs reconstructed to date. GEMs for 5897 bacteria (*light blue*), 127 archaea (*light purple*), and 215 eukaryotes (*pink*) are marked on the phylogenetic tree. Organism names are labeled with *circles* of different colors outside the circular phylogenetic tree, depending on the development methods that were used: manual, Path2Models [83], AGORA [84], and CarveMe [85]. For manually reconstructed GEMs, the relevant PubMed identifier (PMID) or digital object identifier (DOI) for the latest GEM version for an organism is also indicated. The phylogenetic tree was prepared in the same manner as that in Fig. 1. A difference is that a Newick file was created at the species level. A full list of organisms subjected to the GEM reconstruction and used for preparation of phylogenetic trees is available as Additional file 2.
